# Inhibition of lysine methyltransferase G9a/GLP reinstates long-term synaptic plasticity and synaptic tagging/capture by facilitating protein synthesis in the hippocampal CA1 area of APP/PS1 mouse model of Alzheimer’s disease

**DOI:** 10.1186/s40035-021-00247-0

**Published:** 2021-06-29

**Authors:** Javan Lee Tze Han, Karen Ka Lam Pang, Sheila Rui Xia Ang, Mahima Sharma, Sreedharan Sajikumar

**Affiliations:** 1 Department of Physiology, Yong Loo Lin School of Medicine, National University of Singapore, Singapore, 117597 Singapore; 2 Neurobiology Programme, Life Sciences Institute, National University of Singapore, Singapore, 117456 Singapore; 3 Healthy Longevity Translational Research Programme, Yong Loo Lin School of Medicine, National University of Singapore, Singapore, 117456 Singapore

Histone H3 lysine 9 di-methylation (H3K9me2) is an epigenetic repressive histone modification that was found at aberrant states in Alzheimer’s disease (AD) patient samples [1]. The addition of H3K9me2/3 is mainly catalyzed by lysine methyltransferase G9a, which functions as a heteromeric complex with G9a-like protein (GLP). G9a/GLP has other non-histone substrates, including itself. G9a/GLP is implicated in regulating synaptic plasticity, learning and memory [2, 3]. We have previously shown that G9a/GLP inhibition ameliorates exogenous Aß oligomer-induced synaptic plasticity deficits in rat hippocampal slices [3]. In this study, we further tested the effects of G9a/GLP inhibition on synaptic plasticity.

We first tested whether G9a/GLP inhibition could rescue long-term potentiation (LTP) deficits in CA1 area of hippocampal slices of APP/PS1 mice (for materials and methods, see supplemental file). Field excitatory postsynaptic potential (fEPSP) was recorded from the stratum radiatum of CA1 and two stimulating electrodes were used to stimulate two independent Schaffer Collateral inputs (Fig. 1a). We found that a strong tetanization protocol (STET) that typically induces an input-specific late-LTP only induced a transient potentiation (Fig. 1b). When APP/PS1 hippocampal slices were perfused with G9a/GLP inhibitor BIX-01294 (BIX, 500 nM) or UNC-0642 (UNC, 150 nM) during LTP induction by STET, a long-lasting, input-specific potentiation was observed (Fig. 1b). However, when translation inhibitor emetine (20 μM) was co-applied with BIX or UNC, STET only led to a transient potentiation (Fig. 1c). In all recordings, the control untetanized input S2 showed no significant changes in synaptic transmission. These results suggest that G9a/GLP blockade during LTP induction could rescue input-specific synaptic potentiation in APP/PS1 hippocampal slices through upregulating protein synthesis.

**Fig. 1:**
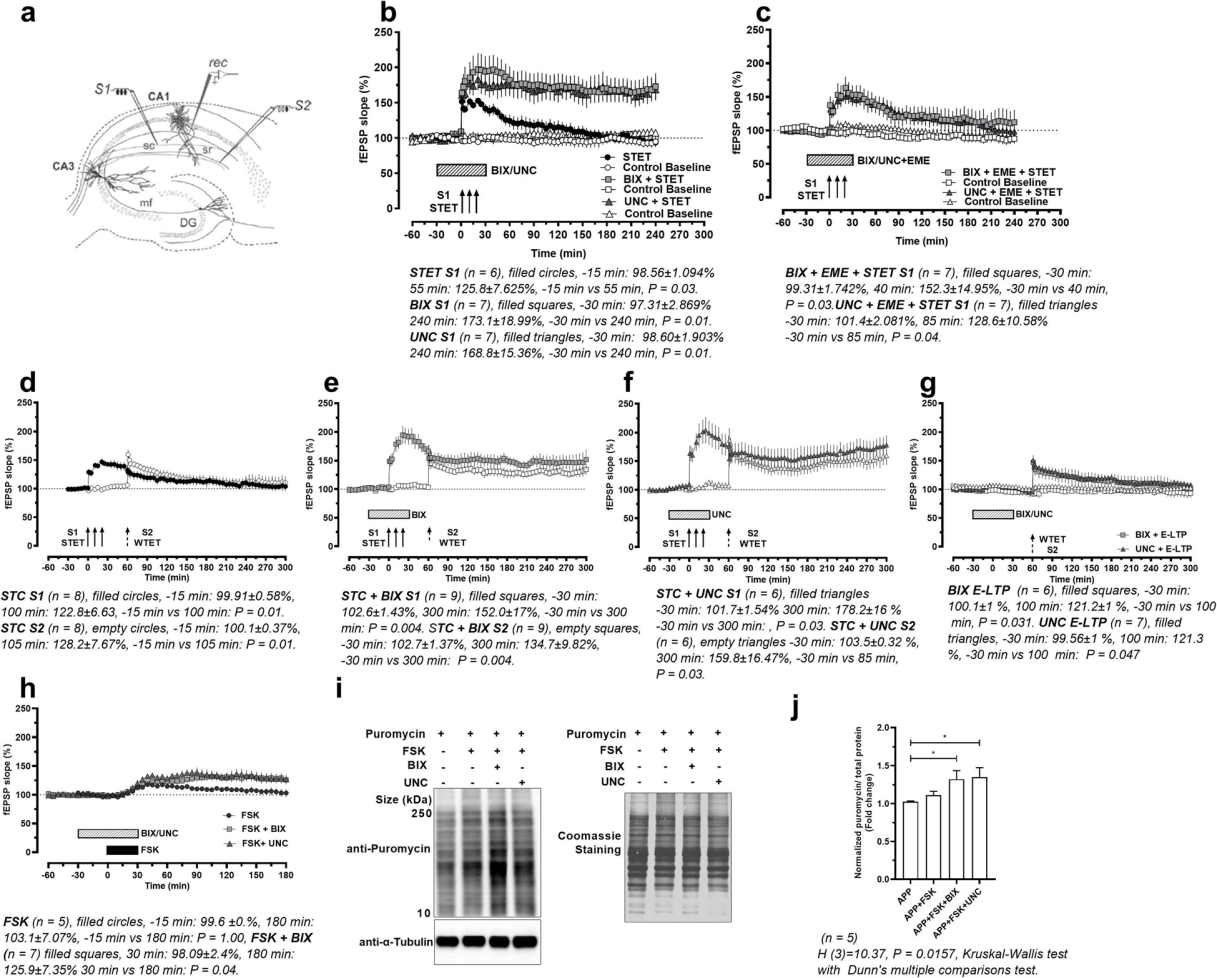
**a** Schematic of in vitro electrophysiological recordings in acute hippocampal slices. **b** LTP in APP/PS1 hippocampal CA1 rescued by BIX/UNC. **c** Emetine (EME) blocked the BIX/UNC-mediated LTP rescue. **d-f** Synaptic tagging and capture was impaired in APP/PS1 hippocampal CA1, but restored by BIX/UNC. **g** Experiments similar as in **d-f** but without STET in S1. **h** Forskolin (FSK)-induced LTP was impaired in APP/PS1 CA1 but rescued by BIX/UNC. Field EPSP values (percentage of baseline) and *P* values of Wilcoxon test at specified timepoints are presented below each figure in **b-h**. **i** SUnSET assay measuring de novo protein synthesis after FSK application with/without BIX/UNC in APP/PS1 slices. **j** Quantification of puromycin in (**i**). All data are presented as mean ± SEM. STC: synaptic tagging and capture; STET: strong tetanization protocol; WTET: weak tetanization protocol

Next, we examined whether G9a/GLP inhibition can restore long-term associative plasticity in hippocampal CA1 of APP/PS1 mice. The synaptic tagging and capture phenomenon is a pathway-specific form of associative plasticity in which a transient, translation-independent LTP (early-LTP) induced by weak stimulation (WTET) is stabilized into a long-lasting LTP (late-LTP) by a strong stimulation (STET) in another independent pathway within a specific time frame [3–5]. In APP/PS1 hippocampal slices, only a transient potentiation was observed after a STET and a WTET were applied 60 min apart to independent synaptic pathways S1 and S2 respectively (Fig. 1d).

Then we perfused APP/PS1 hippocampal slices with G9a/GLP inhibitors (Fig. 1e-f) during STET in synaptic input S1. WTET was subsequently delivered to input S2 30 min after drug application. In both inputs S1 and S2, input-specific potentiation was rescued by G9a/GLP inhibition.

To test whether prior application of G9a/GLP inhibitors alone would transform WTET-induced early-LTP into late-LTP, experiments similar to those in Fig. 1e-f were repeated without late-LTP induction in input S1. After a 30-min washout, only a transient potentiation was observed in input S2 after WTET (Fig. 1g), in line with earlier reports that a WTET only induced a transient early-LTP.

These experiments suggest that acute G9a/GLP inhibition alone cannot induce cell-wide priming and synthesis of plasticity-related products (PRPs) in the AD-like condition. This contradicts prior studies reporting upregulation of synaptic genes and synaptic transmission in Aß-impaired cortical neurons and familial AD (FAD) mice in vivo [1]. This discrepancy may be due to the duration of G9a/GLP inhibitor treatment –acute (1 h) here versus multiple days in other studies, as G9a activity can respond rapidly to external stimuli [6]. Nonetheless, it is also possible that the lack of priming by G9a/GLP inhibition [3] reflects a loss of metaplasticity in APP/PS1 hippocampus [4].

Since G9a/GLP inhibition supported late-LTP maintenance through facilitating protein synthesis in response to plasticity induction, we tested whether G9a/GLP inhibition could also facilitate chemically induced LTP by using the chemical forskolin, an activator of adenylyl cyclase, which is involved in late-phase LTP and AD pathophysiology [7].

Bath application of forskolin (50 μM, 30 min) is typically sufficient to induce potentiation in wild-type hippocampal slices [8]. In contrast, in APP/PS1 slices, forskolin application led to an insignificant increase in fEPSP. When BIX or UNC was co-applied with forskolin, fEPSP remained stably elevated (Fig. 1h). These results suggest that G9a/GLP inhibition could also rescue slow-onset potentiation mediated by adenylyl cyclase activation.

Furthermore, we measured de novo protein synthesis after forskolin application using the SUnSET (surface sensing of translation) assay. Co-application of BIX or UNC with forskolin significantly increased de novo protein synthesis in hippocampal area CA1 (Fig. 1i-j).

Recent studies suggest that high-frequency stimulation-induced LTP and forskolin-induced LTP recruit distinct molecular pathways [9]. Here, G9a/GLP blockade ameliorated deficits in synaptic potentiation induced by both paradigms, suggesting that G9a/GLP mediates fundamental processes underlying synaptic plasticity, and that G9a/GLP is a potential therapeutic target in restoring synaptic function in AD-like conditions.

Open questions remain. Can G9a/GLP inhibition lead to the downregulation of repressive histone mark H3K9me2, thereby upregulating transcription? Are other functions of G9a/GLP, such as methylation of non-histone proteins, altered in AD? More studies are needed to clarify the precise mechanism underlying the G9a/GLP inhibition-induced upregulation of activity-dependent expression of PRPs in the APP/PS1 hippocampus.

## Supplementary Information

**Additional file 1.** Materials and Methods.

## Data Availability

The datasets used and/or analysed during the current study are available from the corresponding author on reasonable request.

## Abbreviations

AD: Alzheimer’s disease
fEPSP: Field excitatory postsynaptic potential
H3K9me2/3: Histone H3 lysine 9 di−/tri-methylation
LTP: Long-term potentiation
PRP: Plasticity-related proteins
