# Supplementary material for: Inhibition of lysine methyltransferase G9a/GLP reinstates long-term synaptic plasticity and synaptic tagging/capture by facilitating protein synthesis in the hippocampal CA1 area of APP/PS1 mouse model of Alzheimer’s disease
Source: Transl Neurodegener. 2021 Jun 29;10:23. doi: 10.1186/s40035-021-00247-0 (PMC8329750; doi:10.1186/s40035-021-00247-0)
Supplement: Supplementary file 1 — Additional file 1. Materials and Methods. [file 40035_2021_247_MOESM1_ESM.docx]

**Supplementary Materials**

#### Materials and Methods

**Animals**

We used a mouse model of AD which expresses a mutated chimeric mouse/human APP and the exon-9–deleted variant of human PS1, both linked to familial AD, under the control of a prion promoter element (*APPSwe/PS1dE9*), which we denote as APP/PS1 (1). Four to six-month-old male transgenic APP/PS1 mice were used. All animals were housed under 12/12 h light/dark cycle and supplied with food and water *ad libitum*.

**Preparation of Acute Hippocampal Slice**

Acute transverse hippocampal slices were prepared as previously described (2). In brief, animals were anesthetized using CO_2_ and decapitated. The brain was immediately removed and transferred to cold (2-4°C) artificial cerebrospinal fluid (ACSF). The ACSF was composed of the following (in mM): 124 NaCl, 3.7 KCl, 1.0 MgSO_4_ ·7H_2_O, 2.5 CaCl_2_ 2H_2_O, 1.2 KH_2_PO_4_, 24.6 NaHCO_3_, and 10 D-glucose, saturated with 95% O_2_–5% CO_2_. Both the left and right hippocampi were isolated in the ACSF, and subsequently, sliced into 400-µm transverse slices with a manual tissue chopper. For field electrophysiology, the slices were quickly transferred to an interface chamber (Scientific System Design, Ontario, Canada) and incubated for at least 2 h at 32 °C with a constant flow of ACSF (1 mL/min) equilibrated with 95% O_2_–5% CO_2_ carbogen (total consumption: 16 L/h). For protein synthesis assay, the slices were transferred to a beaker of oxygenated (95% O_2_–5% CO_2_ carbogen) ACSF maintained at 32 °C in a water bath. The slices were incubated for at least 1.5 h before various drug treatments (see SunSET Protein Synthesis Assay section below for details).

**Electrophysiological Recordings**

A total of 89 hippocampal slices from 45 APP/PS1 mice were used for electrophysiology experiments. Extracellular field recordings were performed on acute hippocampal slices maintained in an interface chamber (Scientific System Design, Ontario, Canada), at 32 °C with a constant flow of ACSF (1 mL/min) equilibrated with 95% O_2_–5% CO_2_ carbogen (total consumption: 16 L/h). Monopolar lacquer-coated stainless-steel electrodes (5MΩ; AM Systems, Sequim, WA, USA) were used for field potential recordings. For one-pathway experiments, a recording electrode was located in the stratum radiatum (SR) of hippocampal area CA1 to record field excitatory postsynaptic potentials (fEPSPs) from the apical dendritic region of area CA1, while a stimulating electrode was placed in the Schaffer collaterals (SC) in the sr of the CA1 area to evoke fEPSP at SC-CA1 synapses. For two-pathway experiments, two stimulating electrodes were placed at equal distances from the recording electrode to stimulate two independent SC inputs to the CA1 pyramidal neurons. Independence of the two synaptic inputs was tested as previously reported (3).

Field potentials were amplified using a differential amplifier (Model 1700, AM Systems, Sequim, WA, USA) and digitized with an analog-to-digital converter (CED 1401, Cambridge Electronic Design, Cambridge, UK). Signals were monitored online and recorded with a custom-made software, PWIN (Leibniz Institute for Neurobiology, Magdeburg, Germany). The software was also used to regulate stimulation.

For each experiment, an input-output curve of stimulus intensity vs fEPSP slope was made and used to determine the basal stimulation intensity. Basal stimulation intensity was set at the value which evoked a fEPSP of 40% of the maximum slope value. Test stimulation comprising four biphasic constant-current pulses (0.2 Hz) was used for recording. A stable baseline was recorded for 30 min or longer before long-term potentiation (LTP) induction or the application of a pharmacological agent. For the experiments in Figures 1A-B, to induce late-LTP, a strong tetanization (STET) protocol comprising three trains of high-frequency stimulation (each train consisted of a single burst of 100 pulses at 100 Hz, 0.2 ms pulse duration; each train was separated by an interval of 10 min) was delivered. For Figure 1C-F, two stimulating electrodes were used to stimulate two independent Schaffer collateral inputs to the same group of CA1 pyramidal neurons. After a 30 min stable baseline, STET was delivered to the first synaptic input S1 to elicit late-LTP. 60 min after the first STET train, a weak tetanization (WTET; single dotted arrow; a single burst of 21 pulses at 100 Hz, 0.2 ms/pulse) was delivered to the second synaptic input S2 to evoke early-LTP. To chemically induce LTP, as in Figure 1G, forskolin (50 µM) was bath applied for 30 min to induce a slow-onset LTP (4).

**Pharmacology**

Two cell-permeable selective inhibitors of the G9a/GLP complex, BIX-01294 (BIX; 270517, Enzo Life Sciences, Singapore) and UNC-0642 (UNC; 5132, Tocris Bioscience, Bristol, UK), were used (5, 6). BIX and UNC were stored as 5 mM and 1 mM stocks in dimethyl sulfoxide (DMSO), respectively. The protein synthesis inhibitor emetine (EME; E2375, Sigma-Aldrich, St. Louis, Missouri, USA) was prepared as 20 mM stock in water. To induce slow-onset chemical LTP, the adenylyl cyclase activator forskolin (FSK; F3917, Sigma-Aldrich, St. Louis, Missouri, USA) was prepaed as 100 mM stock solution in DMSO (4). All stock solutions were stored at -20ºC. BIX, UNC, EME and FSK were diluted in ACSF to final concentrations of 500 nM, 150 nM, 20 µM and 50 µM for bath application for durations as indicated below. The concentration of DMSO in final solutions was maintained below 0.1%, which does not affect basal synaptic responses (7).

**SUnSET Protein Synthesis Assay and Western Blot Assay**

An adapted surface sensing of translation (SUnSET) method was used to end-label newly synthesized proteins with puromycin (8, 9). Mouse hippocampal slices were collected from a total of four groups: APP/PS1 + Puromycin, APP/PS1 + Puromycin + FSK, APP/PS1 + Puromycin + FSK + BIX, APP/PS1 + Puromycin + FSK + UNC. Slices were maintained in a beaker of oxygenated (95% O_2_–5% CO_2_ carbogen) ACSF, at 32 °C, in a water bath. After a 1.5 h recovery period, hippocampal slices were incubated with puromycin (5 µg/ml) 30 min before forskolin treatment and throughout the experiment. For groups that were treated with the G9a/GLP inhibitors, BIX or UNC, the slices were incubated with these inhibitors 30 min before forskolin treatment and for a total of 1 h. Hippocampal slices were treated with forskolin for 30 min and quickly transferred to another beaker with oxygenated ACSF and puromycin for drug washout (Allen et al., 2014). The slices were then collected and flash-frozen in liquid nitrogen 1 h after the end of forskolin treatment.

The CA1 regions of the hippocampal slices were isolated after the SUnSET Protein Synthesis Assay for extraction of total protein. The samples were mechanically homogenized with a 23G needle in Tissue Protein Extraction Reagent (T-PER) (Cat. No: 78510, Thermo Scientific, USA) with HaltTM Protease and Phosphatase Inhibitor Cocktail (Cat. No: 78440, Thermo Scientific, USA). The samples were then incubated on ice for 10 minutes, vortexed for 5 seconds, incubated on ice for another 5 minutes and centrifuged at 10,000 $\times g$, 4°C, for 5 minutes. The supernatant was retained for analysis. Protein concentration was determined using the Bradford protein assay (Cat. No: 500-0205, Quick StartTM Bradford dye reagent, Bio-Rad, USA). After which, 20 µg of the protein extracts were separated on SDS-polyacrylamide gels and transferred to polyvinylidene difluoride (PVDF) transfer membranes (Cat. No: 88518, Thermo Scientific, USA) in a wet transfer cell (Bio-Rad) at 100V for 1.5 h. 5% w/v Bovine Serum Albumin (Cat. No: A7906, Sigma-Aldrich, USA) in TBST was used to block the membranes. Mouse monoclonal anti-puromycin clone 12D10 antibody (1:1000, Cat. No: MABE343, EMD Millipore, USA) and mouse anti-α-tubulin monoclonal antibody (1:50,000, Cat No: T9026, Sigma-Aldrich, Israel) were used to identify puromycin-labeled proteins and α-tubulin, respectively. This was followed by incubation with anti-mouse horseradish peroxidase-conjugated secondary antibody (Cat. No: A4416, Sigma-Aldrich, USA). The immunoproducts were detected using a chemiluminescence detection system according to the manufacturer's instructions (Cat. No: 34580, SuperSignalTM West Pico plus Chemiluminescent Substrate, Thermo Scientific, USA) and developed on a film or using a digital imager (Azure Imager c300). Coomassie Blue staining of the membranes was performed as a loading control staining method. ImageJ (10) was used to quantify the optical density of each protein band. Protein synthesis levels were determined by taking total puromycin-labelled protein lane density in the molecular weight range of 10 to 250 kDa. Each lane of puromycin-labelled protein band density was normalized with its corresponding Coomassie staining lane density. 4-5 hippocampal slices were used for each treatment group, and the experiment was independently repeated five times with 1-2 mice used in each repeat (n = 5, where ‘n’ represents the number of biological replicates).

**Statistical Analyses**

All data were reported as mean±SEM. For electrophysiology data, synaptic efficacy was measured as the slope function of the fEPSP (millivolts per milliseconds). The baseline fEPSP was taken as the fEPSP at -15 min or -30 min, as specified below. To compare fEPSP before-after plasticity induction within a group, fEPSP slopes at specified time points were compared using the Wilcoxon signed rank test (Wilcox test). To compare fEPSP between independent synaptic inputs, the Mann-Whitney test was used. For Western blot data, the nonparametric ANOVA Kruskal-Wallis test was used, followed by Dunn’s multiple comparison test. Statistical significance was taken at *P* < 0.05. Nonparametric tests were used because the small sample sizes of our data did not guarantee a Gaussian distribution (11). All graphs were prepared, and all statistical tests were performed using the GraphPad Prism version 8.0.1 for Windows (GraphPad Software, San Diego, California, USA, www.graphpad.com).

**References**

1. Borchelt DR, Ratovitski T, Van Lare J, Lee MK, Gonzales V, Jenkins NA, et al. Accelerated amyloid deposition in the brains of transgenic mice coexpressing mutant presenilin 1 and amyloid precursor proteins. Neuron. 1997;19(4):939-45.

2. Shetty MS, Sharma M, Hui NS, Dasgupta A, Gopinadhan S, Sajikumar S. Investigation of synaptic tagging/capture and cross-capture using acute hippocampal slices from rodents. Journal of visualized experiments: JoVE. 2015(103).

3. Sajikumar S, Korte M. Metaplasticity governs compartmentalization of synaptic tagging and capture through brain-derived neurotrophic factor (BDNF) and protein kinase Mζ (PKMζ). Proceedings of the National Academy of Sciences. 2011;108(6):2551-6.

4. Allen KD, Gourov AV, Harte C, Gao P, Lee C, Sylvain D, et al. Nucleolar integrity is required for the maintenance of long-term synaptic plasticity. PLoS One. 2014;9(8):e104364.

5. Chang Y, Zhang X, Horton JR, Upadhyay AK, Spannhoff A, Liu J, et al. Structural basis for G9a-like protein lysine methyltransferase inhibition by BIX-01294. Nature structural & molecular biology. 2009;16(3):312-7.

6. Liu F, Chen X, Allali-Hassani A, Quinn AM, Wigle TJ, Wasney GA, et al. Protein lysine methyltransferase G9a inhibitors: design, synthesis, and structure activity relationships of 2, 4-diamino-7-aminoalkoxy-quinazolines. Journal of medicinal chemistry. 2010;53(15):5844-57.

7. Navakkode S, Sajikumar S, Frey JU. Mitogen-activated protein kinase-mediated reinforcement of hippocampal early long-term depression by the type IV-specific phosphodiesterase inhibitor rolipram and its effect on synaptic tagging. Journal of Neuroscience. 2005;25(46):10664-70.

8. Ma T, Trinh MA, Wexler AJ, Bourbon C, Gatti E, Pierre P, et al. Suppression of eIF2α kinases alleviates Alzheimer's disease–related plasticity and memory deficits. Nature neuroscience. 2013;16(9):1299-305.

9. Schmidt EK, Clavarino G, Ceppi M, Pierre P. SUnSET, a nonradioactive method to monitor protein synthesis. Nature methods. 2009;6(4):275-7.

10. Schneider CA, Rasband WS, Eliceiri KW. NIH Image to ImageJ: 25 years of image analysis. Nature methods. 2012;9(7):671-5.

11. Sharma M, Razali NB, Sajikumar S. Inhibition of G9a/GLP complex promotes long-term potentiation and synaptic tagging/capture in hippocampal CA1 pyramidal neurons. Cerebral Cortex. 2017;27(6):3161-71.
